# Glycoside Hydrolase MoGls2 Controls Asexual/Sexual Development, Cell Wall Integrity and Infectious Growth in the Rice Blast Fungus

**DOI:** 10.1371/journal.pone.0162243

**Published:** 2016-09-08

**Authors:** Mengying Li, Xinyu Liu, Zhixi Liu, Yi Sun, Muxing Liu, Xiaoli Wang, Haifeng Zhang, Xiaobo Zheng, Zhengguang Zhang

**Affiliations:** 1 Department of Plant Pathology, College of Plant Protection, Nanjing Agricultural University, and Key Laboratory of Integrated Management of Crop Diseases and Pests, Ministry of Education, Nanjing 210095, China; 2 Agricultural Bureau of Ningxiang County, Changsha 410600, China; Fujian Agriculture and Forestry University, CHINA

## Abstract

*N*-linked glycosylation is a way of glycosylation for newly synthesized protein, which plays a key role in the maturation and transport of proteins. Glycoside hydrolases (GHs) are essential in this process, and are involved in processing of *N*-linked glycoproteins or degradation of carbohydrate structures. Here, we identified and characterized MoGls2 in *Magnaporthe oryzae*, which is a yeast glucosidase II homolog Gls2 and is required for trimming the final glucose in N-linked glycans and normal cell wall synthesis. Target deletion of *MoGLS2* in *M*. *oryzae* resulted in a reduced mycelial growth, an increased conidial production, delayed conidial germination and loss the ability of sexual reproduction. Pathogenicity assays revealed that the Δ*Mogls2* mutant showed significantly decreased in virulence and infectious growth. Further studies showed that the mutant was less sensitive to salt and osmotic stress, and increased sensitivity to cell wall stresses. Additionally, the Δ*Mogls2* mutant showed a defect in cell wall integrity. Our results indicate that MoGls2 is a key protein for the growth and development of *M*. *oryzae*, involving in the regulation of asexual/sexual development, stress response, cell wall integrity and infectious growth.

## Introduction

Rice blast, also known as rice fever, is a devastating disease of rice caused by *Magnaporthe oryzae*, and has occurred in most rice-producing areas worldwide [1]. In appropriate environmental conditions, the disease can spread quickly and lead to 10–20% rice yield losses [1,2]. The fungal pathogen initiates infection by attachment of conidia to the plant surface and formed a dome shaped structure called an appressorium at the germ tube tip [3]. The mature appressorium generates huge turgor that forces invasive nail through rice epidermis into the interior of plant cells, thus causing diseases [4,5]. However, identification and characterization of pathogenicity-associated genes in *M*. *oryzae* will help us to better understand the molecular mechanisms of the pathogen, and also will benefit the development of new disease management strategies.

*N*-linked glycosylation is a process of ubiquitous protein modification in eukaryotic cells, and results in a systemic modification of the proteome [6]. It is also an essential post-translational modification of secretory and membrane proteins in all eukaryotes, which is carried out in endoplasmic reticulum (ER) [7]. Protein *N*-glycosylation occurs by transfer of a specific oligosaccharide (Glc3Man9GlcNAc2; G3) from a dolichol donor to asparagine (Asn) residues in the Asn-Xaa-(Ser/Thr) sequence of nascent polypeptide chains. The transferred oligosaccharide is then processed in the ER by the sequential action of specialized trimming enzymes. Glucosidase I (Gls1) removes the outermost 1,2-linked glucose residue to yield the G2 form of the glycan (Glc2Man9GlcNAc2) [8], then glucosidase II (Gls2) removes the middle and innermost 1,3-linked glucose residues, yielding the G1 and M9 forms (Glc1Man9GlcNAc2; Man9GlcNAc2), respectively. Finally, mannosidase I selectively removes a specific mannose residue, yielding the M8 form (Man8GlcNAc2). Then glycoproteins either exit in the ER or are targeted for degradation if they have failed to fold correctly [9]. In this process, glycoside hydrolases are essential proteins and involved in processing of *N*-linked glycoproteins or degradation of carbohydrate structures.

Glycoside hydrolases are various enzymes making general glycosidic bond hydrolysis of glycoside or oligosaccharides[10]. α-glucosidase II belongs to glycoside hydrolase 31 family, and is a heterodimer composed of α subunit, responsible for the catalytic activity, and *β* subunit that retains the complex within the ER lumen though a KDEL type ER retention signal [11–13]. Alpha subunit is a 95–110 kDa protein conserved in fungi, animals, and plants that contains the consensus sequence (G/F)(L/I/V/M)WXDMNE) that is the active site of glycoside hydrolase 31 family [13]. When the catalytic α subunit complexed with two different glucosyl ligands containing the scissile bonds of first and second-step reactions, non-reducing terminal disaccharide moieties of the two kinds of substrates can be accommodated in a gourd-shaped bilocular pocket, thereby providing a structural basis for substrate-binding specificity in the two-step deglucosylation catalyzed by this enzyme [14]. Gls2 plays a key role in quality control of glycoprotein folding in ER, andis also responsible for the removal of the glucose added by UGGT. Cycles of deglucosylation and reglucosylation catalyzed by the opposing activities of UGGT and Gls2 continue until the glycoproteins acquire their native structure [15].

Alpha-glucosidase II is essential for metabolism of carbohydrates, especially in glycoprotein processing [16]. In fungi, defects in the glucosidases lead to changes in the cell wall composition and organization, activation of the cell integrity pathway. In *Saccharomyces cerevisiae*, cell wall 1,6-*β*-glucan synthesis depends on ER glucosidases I and II [17]. In *Trichoderma reesei*, a frameshift mutation of glucosidases II results in the aberrant glycosylation profile [18]. In fungal pathogens, defects in the protein glycosylation pathways lead to attenuation of virulence, delayed dimorphism, and defects in the interaction with the host immune system. In *Candida albicans*, α-glycosidases are required for *N*-glycosylation, cell wall integrity, and normal host-fungus interaction [9]. In *Ustilago maydis*, ER glucosidases and protein quality control factors are essential in development and infection [19]. While in *S*. *pombe*, Gls2α mutants accumulate misfolded glycoproteins in the ER under nonstressed conditions and shows undiscernible phenotype[13]. However, *N*-linked glycosylation and its associated glycoside hydrolases in *M*. *oryzae* have not been well studied yet. Only Chen *et al* found that *α*-1,3-mannosyltransferase in the rice blast fungus was required for *N*-glycosylation of effectors and suppress host innate immunity [20]. In this study, we identified and characterized a *α*-glucosidase *α* subunit homolog MoGls2 in *M*. *oryzae*, and found MoGls2 plays pleotropic roles in the growth and development of the pathogen.

## Materials and Methods

### Strains and culture conditions

The *M*. *oryzae* Guy11 strain was used as the wild type in this study. All strains described in this study were cultured on complete medium (CM) agar plates at 28°C. Fungal mycelia were harvested from liquid CM and used for genomic DNA and RNA extraction. *M*. *oryzae* transformation was performed as described previously [21]. For vegetative growth, mycelial plugs (3 mm×3 mm) were placed onto CM, MM, OM and SDC medium and cultured at 28°C for 7 days [22]. For stress response assay, indicated strains were inoculated onto CM plates containing 0.7 M NaCl, 0.6 M KCl, 1 M sorbitol, 200 μg/ml CFW, 400 μg/ml CR and 0.01% SDS, respectively. Colony diameter was measured 7 days after culture at 28°C, and the inhibition rate was analyzed as previously described [23]. All experiments were repeated three times, with three replicates each time.

### *MoGLS2* gene deletion and complementation

To make the gene deletion construct, a 1-kb upstream and a 1-kb downstream flanking sequence fragment of *MoGLS2* was amplified from *M*. *oryzae* genomic DNA by PCR. Two fragments were cloned into pCX62 vector flanking hygromycin phosphotransferase gene (*HPH*) to get pCX62::*MoGLS2*::*HPH*. Then, the sequenced pCX62::*MoGLS2*::*HPH* plasmid was used as template to amplify a 3.4-kb fragment by primers MGG_08623F1 (F)/MGG_08623F4 (R). The final 3.4-kb fragment was used for protoplast transformation of *M*. *oryzae*. The complemented fragment containing the entire *MoGLS2* gene and its native promoter region was amplified by PCR and then co-transformed into yeast competent cells XK-125 with the pYF11 vector that linearized by *Xho* I to constitute pYF11::*MoGLS2* construct to complement the mutant strain. Primers used in this section are listed in S1 Table.

### Conidiation, appressorium formation and turgor measurement assays

Mycelial plugs (3 mm×3 mm) of Guy11, Δ*Mogls2* mutant and the complemented transformant Δ*Mogls2*/*MoGLS2* were inoculated onto SDC plate, cultured at 28°C in darkness for 7 days, and then kept under a black light for 3 days to induce conidiation. Conidia were collected with distilled water and counted using a hemacytometer under a microscope. For appressorium formation assay, a 25 μl conidial suspension with the concentration of 1×10^4^ spores/ml was dropped on coverslips, and cultured at 28°C for 2, 4, and 6 h [24]. Appressorium turgor was measured as previously described [23]. All experiments were repeated three times, with three replicates each time.

### Sexual reproduction assay

Mycelial plugs of Guy11, Δ*Mogls2* mutant and Δ*Mogls2*/*MoGLS2* with TH3 strain were inoculated 3 cm apart on OM plates, respectively. The plates were cultured at 20°C with continuous light for 3–4 weeks and perithecia production was evaluated. Ascus was observed under a microscope to calculate the number of mature perithecia and ascospores. The experiment was repeated three times, with three replicates each time.

### Pathogenicity assays

Pathogenicity was tested on rice and barley leaves. Plant spraying assays were performed as described [25]. For detached leaf assay, conidia suspensions of different concentrations (10^5^, 10^4^ and 10^3^ spores/ml) were inoculated onto detached barely leaves. Diseased leaves were photographed at 5 (barley) or 7 (rice) days post-inoculation (dpi). Rice leaf sheath assay was performed by inoculating with 100 μl of conidial suspension (5×10^4^ spores/ml) on the inner leaf sheath cuticle cells. Infectious growth was observed at 32 hours post-inoculation (hpi) under humid conditions at 28°C. Each type of infectious hypha was observed under a microscope and the infection was statistically analyzed. All experiments were repeated three times, with three replicates each time.

### Quantitative real-time PCR assay

Total RNA samples were isolated from vegetative hyphae of Guy11 and the *ΔMogls2* mutant cultured in liquid CM for 2 days, and used for cDNA synthesis with the HiScript Q Select RT SuperMix for qPCR kit (Vazyme Biotech, Nanjing, China) following the instructions. The RT2 PCR Real-Time SYBR Green/ROX PCR master mix (TaKaRa, Dalian, China) was used for qRT-PCR analysis. The relative quantification of each transcript was calculated by the 2^-ΔΔCT^ method [26] with the *M*. *oryzae ACTIN* gene as the internal control. For each gene, qRT-PCR assay repeated three times with three biological replicates.

### Protoplast production assay

The wild type Guy11, Δ*Mogls2* mutant and Δ*Mogls2*/*MoGLS2* were cultured in liquid CM media for 2 days and the mycelia were collected by centrifugation for 10 min at 5,000 rpm. The following lysis and protoplast release steps were performed as described previously [27]. The cell wall degradation and the number of protoplasts were examined under a microscope and counted using a hemacytometer with three replicates. The experiment was repeated three times, with three replicates each time.

## Results

### Identification of MoGls2 in *M*. *oryzae*

**Fig 1:**
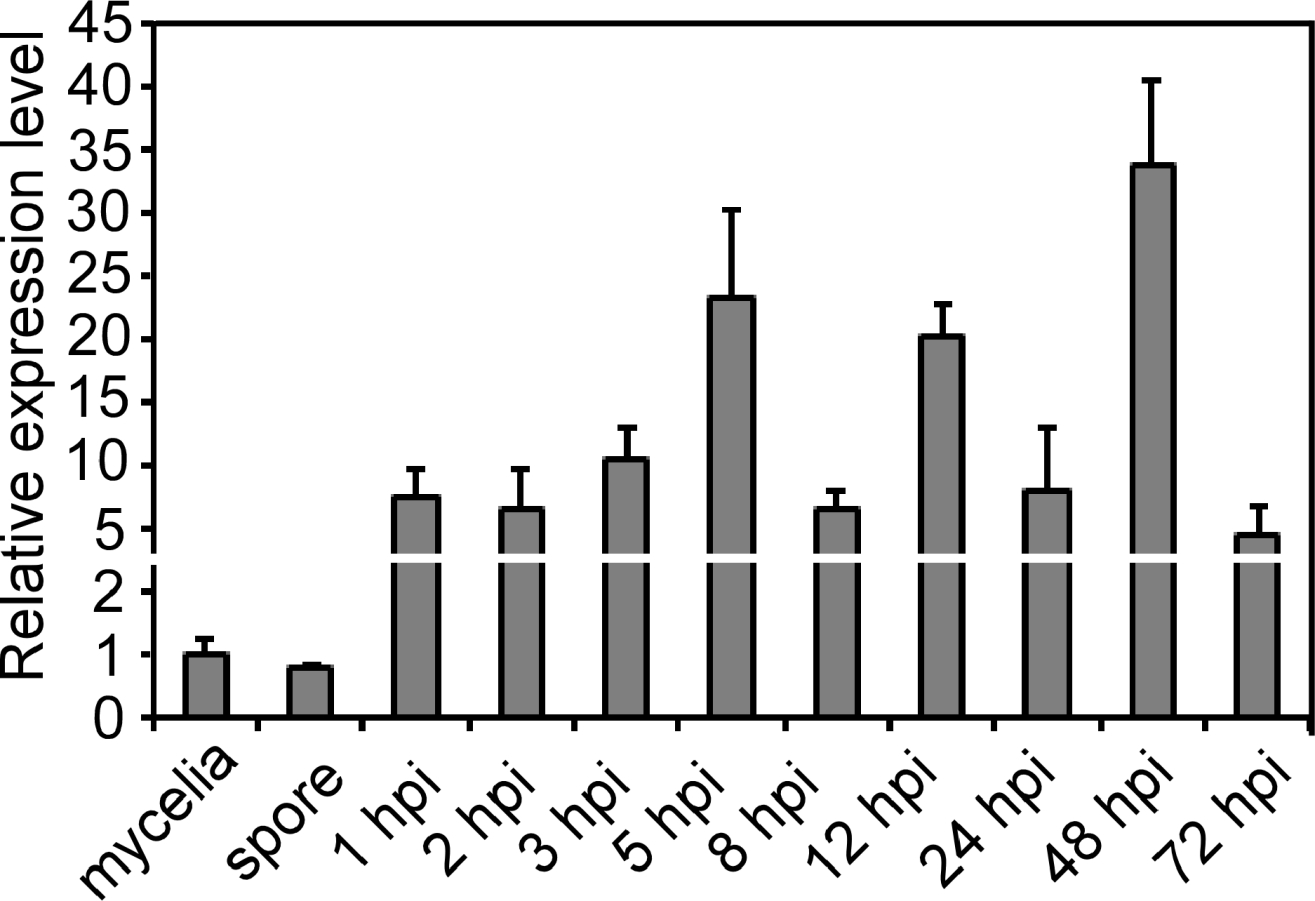
Expression profiles of *MoGLS2* during fungal developmental stages. RNA was extracted from mycelia, conidia and infectious stages, respectively. *ACTIN* was used for normalization and values represent mean ± SD from three independent experiments each with three replicates.

Using the yeast Gls2 protein sequence to perform BLAST_P comparison in *M*. *oryzae* genome database, a homolog (MGG_08623) that named MoGls2 was identified. *MoGLS2* encodes a 980 amino acids protein with no intron. The mature protein contains a signal peptide, a galactose mutarotase domain at N-terminus and a glycoside hydrolase domain in C-terminus (S1A Fig). Phylogenetic analysis MoGls2 and its homologs revealed that Gls2 proteins were well conserved in different fungi, and MoGls2 showed high amino acid identity with Gls2 from *Aspergillus oryzae* (59%), *Trichoderma reesei* (64%), *Fusarium oxysporum* (76%), *Beauveria bassiana* (64%), *Verticillium dahlia* (68%), *Neurospora crassa* (67%), *Penicillium roqueforti* (58%), *Schizosaccharomyces pombe* (45%), *Saccharomyces cerevisiae* (38%) and *Gaeumannomyces graminis* (78%) (S1B Fig). Transcription profile analysis of *MoGLS2* during different developmental stages of *M*. *oryzae* showed that *MoGLS2* was highly expressed during infection but not during conidiation when compared to the mycelial stage. The transcription level was increased 5- to 33-fold during infection stages when compared to the mycelial stage (Fig 1), indicating that *MoGLS2* likely have a potential role during the infection process of the rice blast fungus.

### Generation of *MoGLS2* gene deletion mutant and complemented transformant

To investigate the biological role of *MoGLS2* in *M*. *oryzae*, a knockout vector with hygromycin phosphotransferase gene (*HPH*) to replace *MoGLS2* through homologous recombination was constructed (S2A Fig). The final pCX62::*MoGLS2*::*HPH* vector was transformed into wild type Guy11 using PEG-mediated protoplast transformation approach. The resulting hygromycin resistant transformants were screened by PCR and further confirmed by Southern blot analysis to obtain the Δ*Mogls2* mutants. The result revealed that *MoGLS2* gene was successfully deleted in the mutant with a single copy in the *M*. *oryzae* genome (S2B Fig). The complemented transformant was obtained by transforming the construct pYF11::*MoGLS2*::GFP into protoplast of the Δ*Mogls2* mutant. The phenotypes of the wild type Guy11, Δ*Mogls2* mutant and the complemented transformant Δ*Mogls2/MoGLS2* were analyzed.

### MoGls2 is involved in vegetative growth

**Fig 2:**
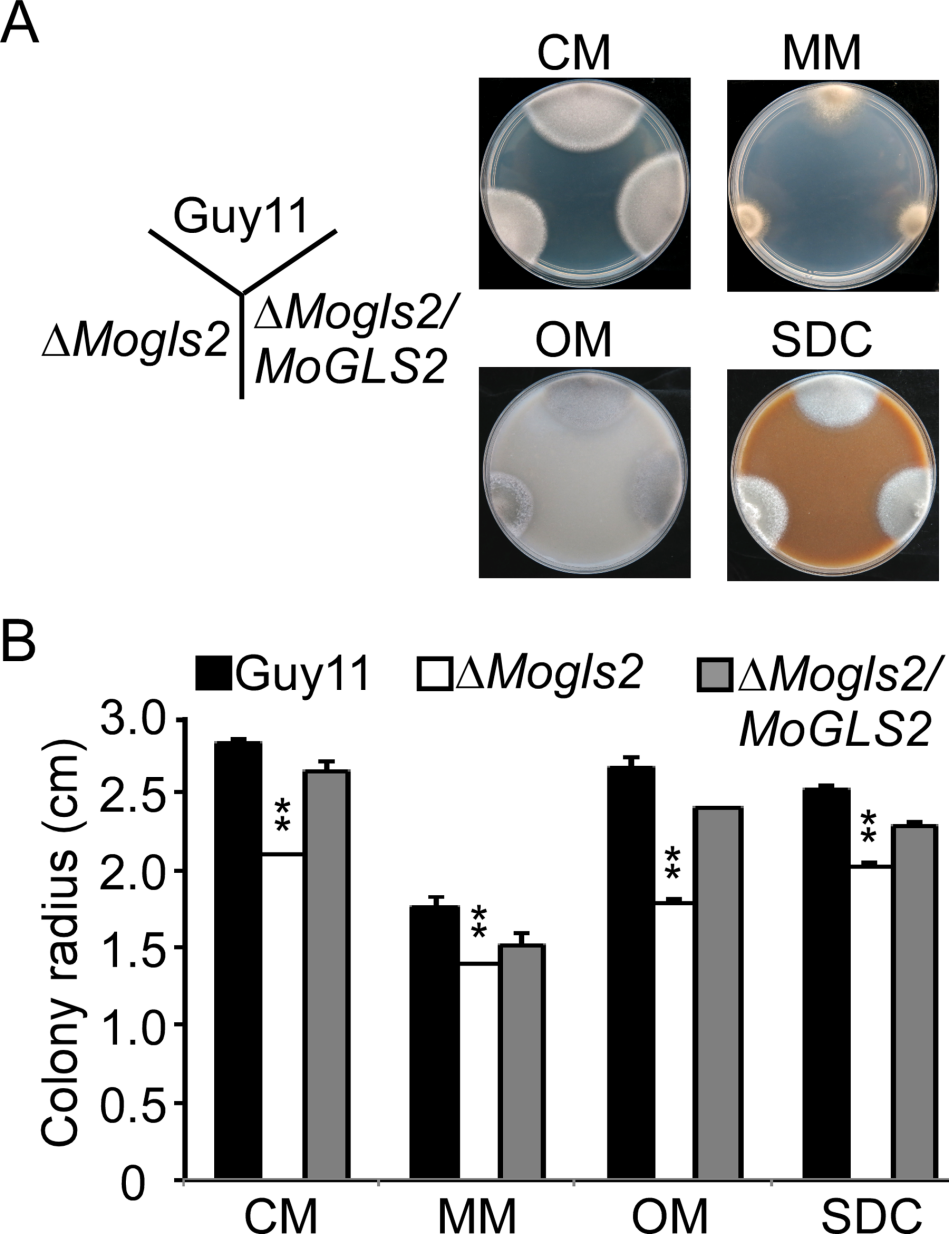
Colony morphology and growth rate of the *ΔMogls2* mutant on various media. (A) Colony morphology was observed on CM, MM, OM and SDC ager plates, and photographed after culture at 28°C in darkness for 7 days. (B) The colony radius were measured and subjected to statistical analysis. Error bars are standard deviations and asterisks represent significant differences with *P*<0.01(**).

We first observed the morphology and test the growth rate of the Δ*Mogls2* mutant on CM agar plates. The Δ*Mogls2* mutant displayed a significantly smaller colony and a slower growth rate in comparison to the wild type strain and the complemented transformant (Fig 2A and 2B). To address whether the mutant was nutrient-dependent, the indicated strains were further inoculated onto MM, OM and SDC agar plates. Compared to Guy11 and the complemented transformant, the growth rate of the Δ*Mogls2* mutant was also significantly decreased. However, the colony morphology, such as aerial hyphae and hyphae branch of the mutant on these four media was not apparently changed (Fig 2A and 2B). These results indicated that MoGls2 plays a role in vegetative growth in the rice blast fungus.

### MoGls2 plays critical roles in asexual and sexual development

**Fig 3:**
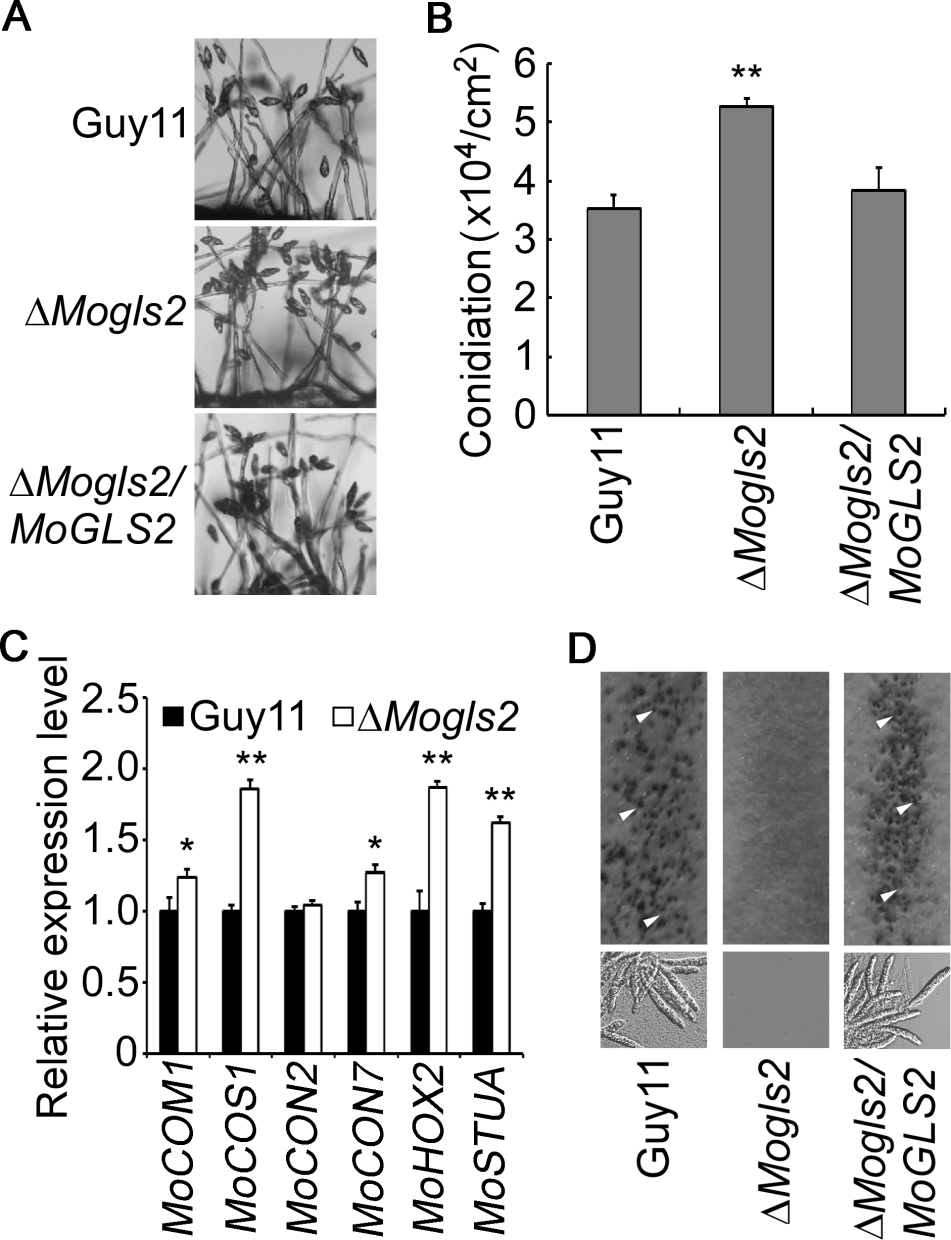
Asexual and sexual development of the *ΔMogls2* mutant. (A) Conidial development was observed under a light microscope 20 h after induction of conidiation on cover slips. (B) Statistical analysis of the production of conidia. (C) qRT-PCR analysis the expression of several conidiation-related genes in the wild-type and *ΔMogls2* mutant. Error bars are standard deviations and asterisks represent significant differences with *P*<0.01(**) or *P*<0.05(*). (D) Perithecia and asci developed by wild type and *ΔMogls2* mutant were photographed three weeks after inoculation. Arrows indicate peritheria.

Asexual spores are important for *M*. *oryzae* to complete its disease cycle. Therefore, wild type Guy11, Δ*Mogls2* mutant and the complemented transfromant were inoculated onto SDC plates to induce conidial production. We first observed the conidiophore and conidia induced on glass slides, and found the Δ*Mogls2* mutant formed normal conidiophores but with more conidia compared to the wild type (Fig 3A). We further quantified the conidial number of the indicated strains cultured for 10 days on SDC plates. Consistent to the result on glass slides, the conidial production was significantly increased in the Δ*Mogls2* mutant, and was 1.5-fold of that the wild type (Fig 3B). To address how MoGls2 controls the conidial production, the expression of six genes (i.e. *MoCON1*, *MoCOS1*, *MoCON2*, *MoCON7*, *MoHOX2* and *MoSTUA*) that involved in conidiation was evaluated. The results showed that *MoCOS1*, *MoHOX2* and *MoSTUA* were significantly up-regulated in the Δ*Mogls2* mutant (Fig 3C), indicating MoGls2 controls conidiation through negative regulating the expression of *MoCOS1*, *MoHOX2* and *MoSTUA* in *M*. *oryzae*. We also analyzed the sexual development of the Δ*Mogls2* mutant, no perithecia was formed by the mutant and the mutant completely lost the ability to produce sexual progeny. In contrast, wild type and the complemented transformant produced numerous mature perithecia under the same conditions (Fig 3D). We conclude that MoGls2 plays a crucial role in asexual development and an essential role in sexual development in *M*. *oryzae*.

### MoGls2 is important for penetration and infectious growth

**Fig 4:**
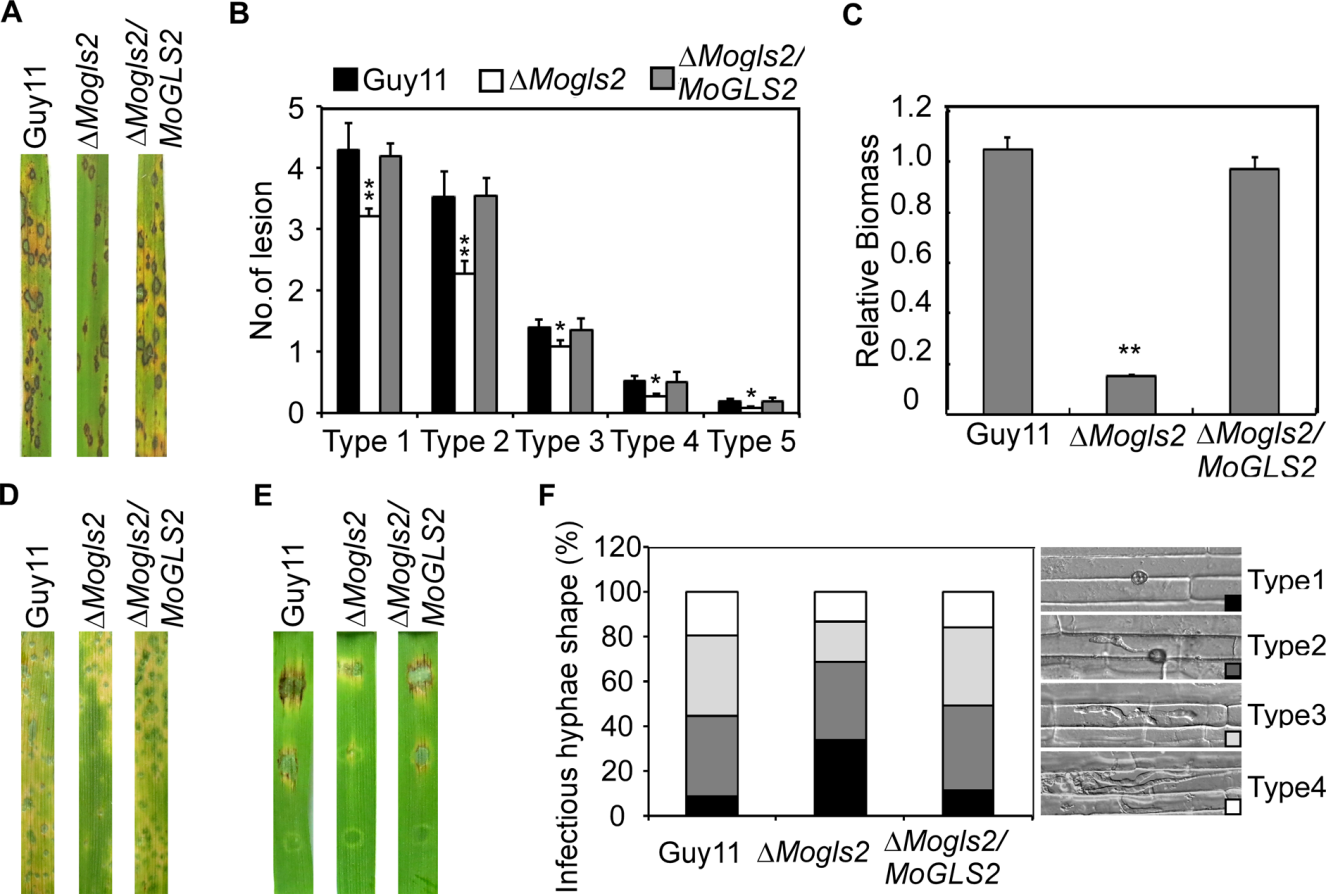
Disease symptoms and infectious growth of the *ΔMogls2* mutant. (A) Two-week-old rice seedlings were sprayed with conidial suspensions (5×10^4^ spores / ml) of wild type, mutant and the complemented transformant. The disease symptoms were observed and photographed at 7 days post inoculation (dpi). (B) Statistical analysis of the number of lesions in each type. Ten pieces of diseased rice leaves were counted per replicate and the experiment was repeated three times. (C) qRT-PCR analysis of the fungal biomass in the diseased rice leaves. (D) One-week-old barley seedlings were sprayed with conidial suspensions (5×10^4^ spores/ml) from the indicated strains. The symptoms were observed and photographed at 5 dpi. (E) Conidia (1×10^5^ spores/ml, 1×10^4^ spores/ml and 1×10^3^ spores/ml) were dropped onto detached barley leaves and the infected leaves were photographed at 5 dpi. (F) Statistical analysis of the percentage of infectious hyphae type in the rice sheath cells (type 1: no infectious hyphae; type 2: one infectious hyphae; type 3: two or three branches restricted in one cell; type 4: more than three branches extended to neighboring cell. N = 50).

To investigate whether MoGls2 contributes to virulence, conidial suspensions of Guy11, Δ*Mogls2* mutant and the complemented transfromant were sprayed onto the rice seedlings, respectively. After 7 days inoculation, the mutant showed reduced virulence with less and smaller lesions in comparison to numerous typical lesions caused by the wild type Guy11 and the complemented transfromant (Fig 4A). Further lesion-type scoring assay revealed that the number of lesions caused by Δ*Mogls2* mutant was significantly decreased (Fig 4B) [28]. The diseased leaves inoculated by Δ*Mogls2*mutant also showed a reduction in fungal biomass (Fig 4C). We also performed spraying assay and detached leaf assay on barley seedlings, and both assays showed similar results to that on rice leaves (Fig 4D and 4E). Because the Δ*Mogls2* mutant caused less and smaller lesions, penetration and infectious hyphal growth was observed in the rice sheath cells. Over 30% appressoria of the Δ*Mogls2* mutant were unable to penetrate through the plant cells (type 1). 53% infectious hyphae of the Δ*Mogls2* mutant restricted in one cell with no or with 2–3 branches (type 2 and type 3) and less than 15% extended to neighboring cells with more branches (type 4). In contrast, less than 10% of type 1 and over 50% of type 3 and type 4 was observed in the wild type Guy11 and the complemented transformant (Fig 4F). These results implicated that MoGls1 plays a critical role in penetration and infectious growth in the rice blast fungus.

### MoGls2 plays a role in conidial germination but not appressorial turgor pressure

**Fig 5:**
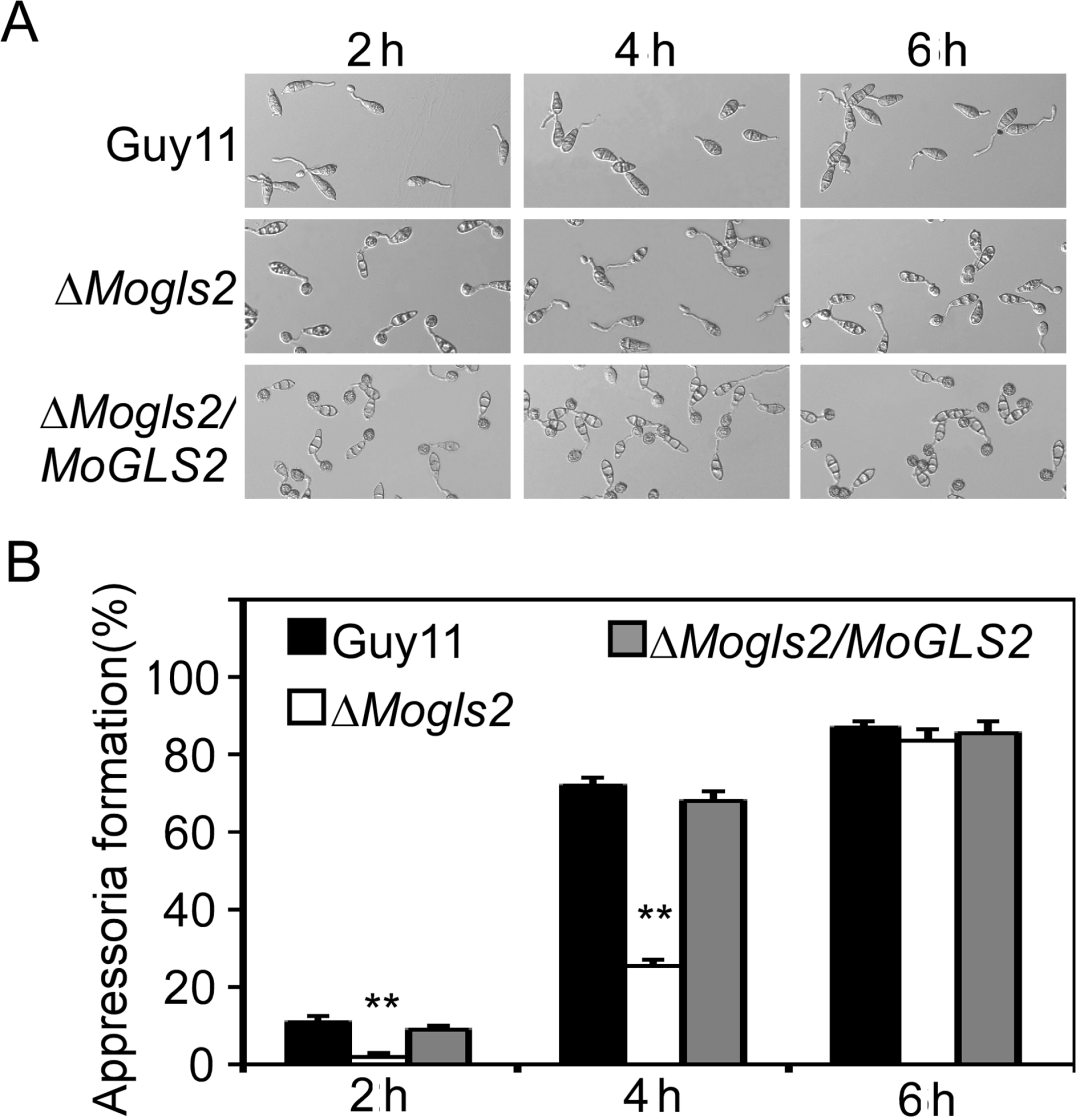
Conidial germination and appressorium formation of the *ΔMogls2* mutant. (A) **Conidial germination** and appressorium formation was observed at 2, 4 and 6 h on hydrophobic surfaces under a microscope. (B) Statistical analysis of the percentage of appressorium formation at indicated time courses. Error bars are standard deviations and asterisks represent significant differences with *P*<0.01(**). The experiment was replicated three times.

Appressoria are critical structures for infection of *M*. *oryzae*. To clarify whether the infection defects of Δ*Mogls2* mutant due to appressoria, conidia were allowed to germinate and form appressoriaon inductive surface at time courses. The result showed that conidial germination was delayed in the mutant at early stage compared to the wild type Guy11 and the complemented transformant. At 2 h post-inoculaton (hpi), 55% conidia have germ tubes in the mutant, compared to 80% in the wild type. At 4 hpi, conidial germination increased to 97% in both mutant and wild type (Fig 5A). Consistent with this, appressorial formation was also delayed in the mutant, 2% conidia of the mutant formed appressoria, compared to 11% in the wild type at 2 hpi, and increased to 25% in the mutant and 72% in the wild type at 4 hpi. While over 83% conidia of both wild type and mutant formed appressoria at 6 hpi (Fig 5A and 5B). This result indicated that MoGls2 has a role in conidial germination. The delaying conidial germination resulted in the delaying appressorial formation of the mutant. Appressorium turgor was indispensable for normal function of appressoria. Therefore, appressorium turgor was measured using an incipient cytorrhysis (cell collapse) assay. However, percentage of collapsed appressoria of Δ*Mogls2* mutant showed no significant difference compared to the wild type and the complemented transformant (S3A Fig). We further monitored glycogen accumulation during appressorial maturation, and found no significant difference between the mutant and wild type (S3B Fig), indicating MoGls2 does not play an essential role in appressorium turgor generation.

### MoGls2 is involved in stress response

**Fig 6:**
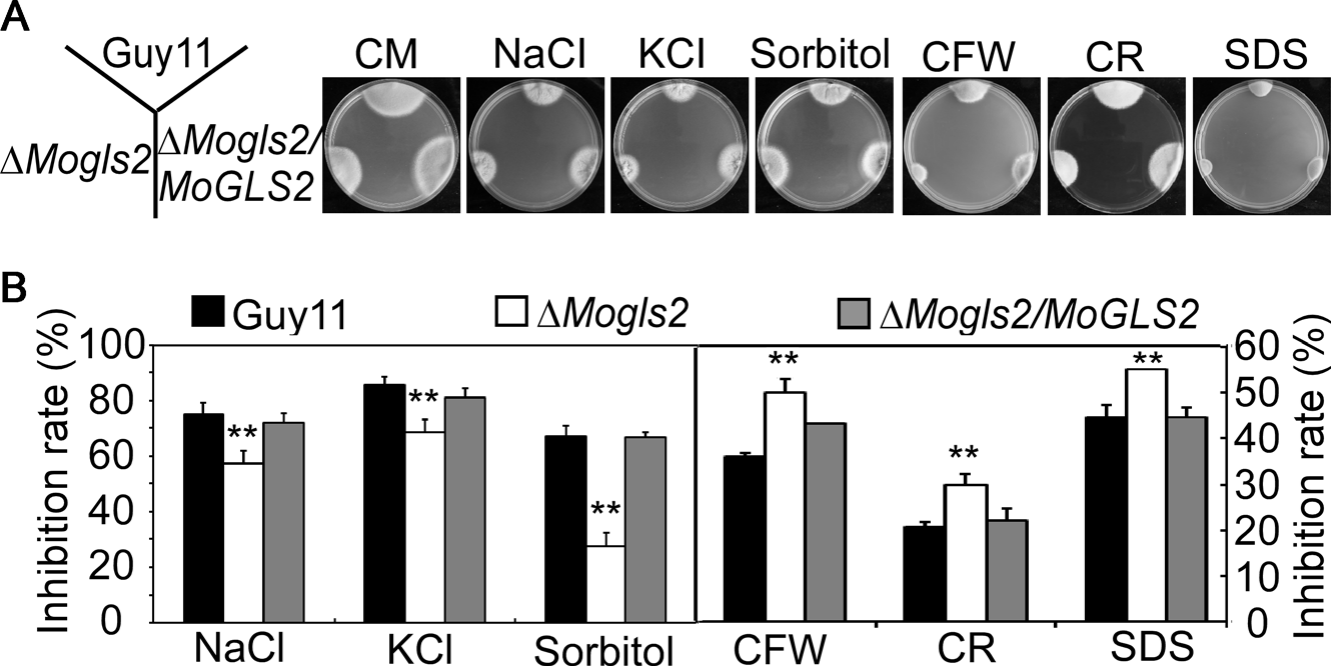
Stress response assays of the Δ*Mogls2* mutant. (A) Wide type Guy11, *ΔMogls2* mutant and the complemented transformant were inoculated on CM plates containing NaCl, KCl, sorbitol, CFW, SDS and Congo red (CR), respectively. Plates were photographed at 7 dpi. (B) Statistical analysis of the inhibition rate of the indicated strains exposed to different stresses. Error bars are standard deviations and asterisks represent significant differences with *P*<0.01(**).

Adaption to various stress conditions affect the normal growth and development of *M*. *oryzae* and the ability to infect the host [29]. To investigate whether MoGls2 participates in external stress response, wild type Guy11, Δ*Mogls2* mutant and the complemented transformant were inoculated onto CM plates containing salt stresses (NaCl and KCl), osmotic stress (sorbitol) and cell wall stress (CFW, SDS and Congo red), respectively. Colony diameter and inhibition rate were analyzed 7 days after cultured at 28°C. The results showed that the Δ*Mogls2* mutant was less sensitive to salt and osmotic stresses. Compared to the wild type, inhibition rate of the mutant was decreased 12% (NaCl), 8.4% (KCl) and 19.9% (Sorbitol), respectively (Fig 6A and 6B). In contrast, the Δ*Mogls2* mutant displayed increased sensitivity to cell wall stresses and the inhibition rate was increased 14% (CFW), 9% (CR) and 10% (SDS), respectively (Fig 6A and 6B). These results indicated that MoGls2 has a role in response and adaptation to various stresses of *M*. *oryzae*.

### MoGls2 is important for maintenance of cell wall integrity

**Fig 7:**
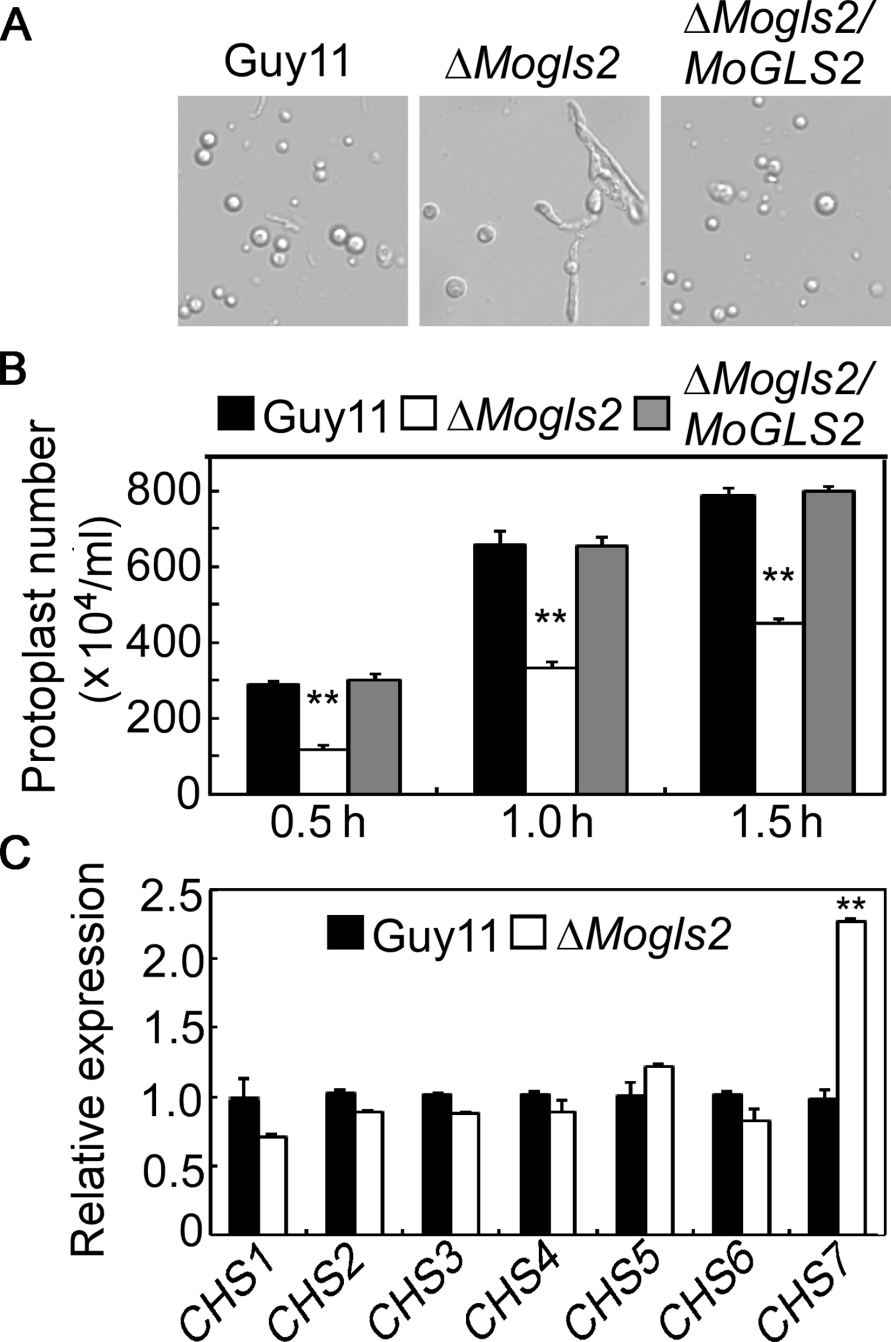
Assays for the defects of the Δ*Mogls2* mutant in cell wall integrity. (A) Light microscopic examination of protoplast release after cell wall degradation enzyme treatment for 60 min and photographed. (B) Quantification of protoplast production of Guy11 and the Δ*Mogls2* mutant treated by cell wall degrading enzyme. (C) qRT-PCR analysis of the expression of several chitin synthase genes in the wild-type and Δ*Mogls2* mutant. Error bars are standard deviations and asterisks indicate a significant difference between the mutant and wild-type strain with *P<0*.*01* (**).

Because the Δ*Mogls2* mutant has defects in response to salt, osmotic and cell wall stresses, implicating it might be defective in cell wall integrity. Thus, we further observed the chitin contribution in hyphae and assayed whether the mutant sensitive to cell wall degradation enzyme (lysing) by quantification the released protoplast in the lysing solution. The result revealed that the chitin contribution in the Δ*Mogls2* mutant showed no obviously difference compared to the wild type (S4A Fig). However, the mutant was less sensitive to lysing and many hyphal fragment remainings could be observed in the lysing cultures at 1 hour post-incubation (hpi). In contrast, a large number of protoplasts with few hyphal fragments were observed in that of wild type and the complemented transformant under the same conditions (Fig 7A). The number of protoplasts produced by Δ*Mogls2* mutant decreased to 40.5, 50.4 and 57.2% of that wild type at 0.5, 1 and 1.5 hpi, respectively (Fig 7B), indicating the mutant has a defect in cell wall integrity. Since chitin is the main component of fungal cell wall, we further analyzed the expression of seven chitin synthase encoding genes (*CHS1*-*7*). The result showed that only *CHS7* was up-regulated in the Δ*Mogls2* mutant (Fig 7C). However, chitin content of the mutant showed no significant change compared to the wild type (S4B Fig). These results indicated that MoGls2 plays a role in cell wall integrity in the rice blast fungus.

### The signal peptide is essential for the function but not proper localization of MoGls2

**Fig 8:**
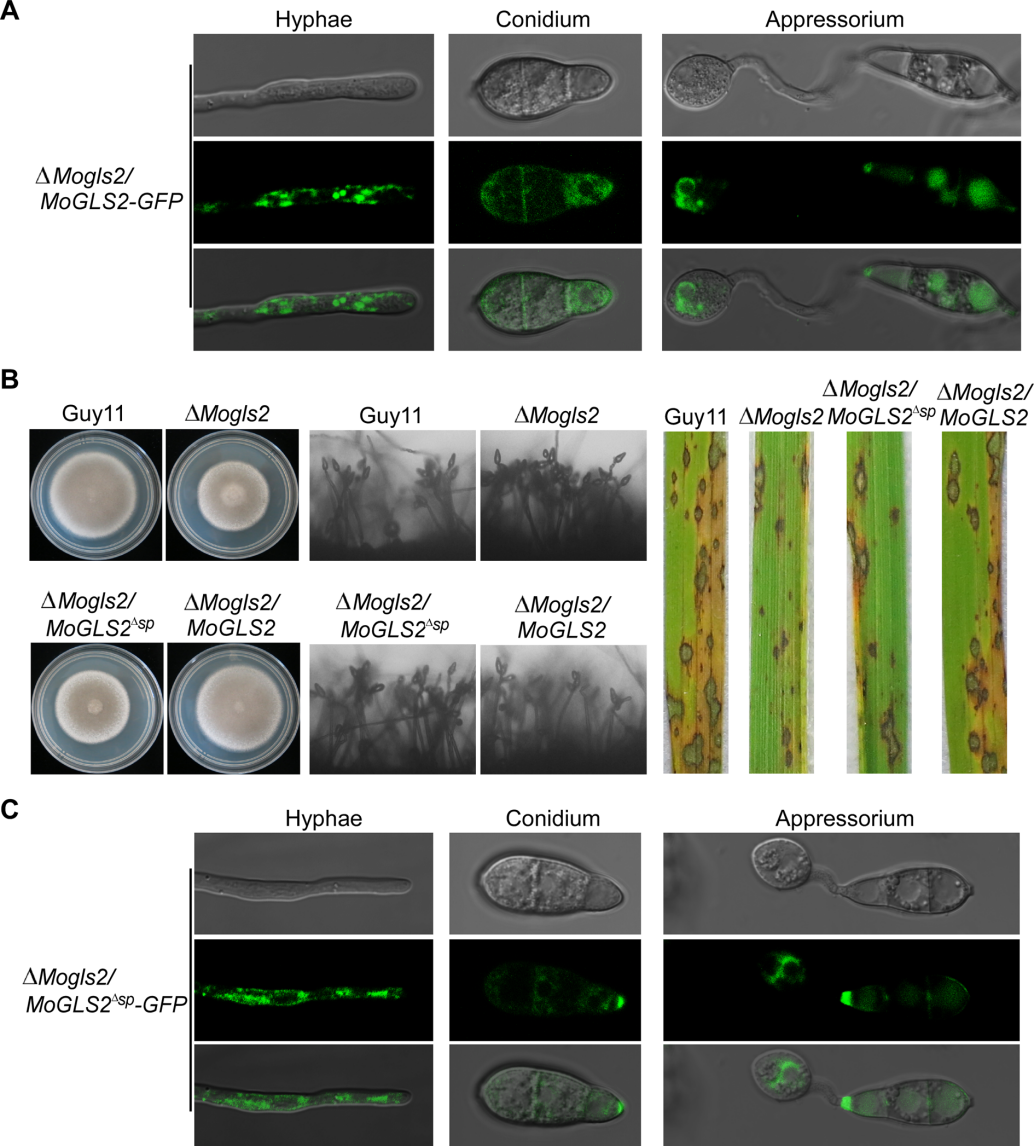
Cellular localization of MoGls2 and phenotype analysis of the Δ*Mogls2/MoGLS2*^ΔSP^ transformant. (A) Vegetative hyphae, conidium and appressorium of the Δ*Mogls2/MoGLS2*^ΔSP^ transformant was observed under a fluorescence microscope. (B) Vegetative growth, conidiation, and infection assays of the indicated strains. (C) GFP fluorescence observation of the Δ*Mogls2/MoGLS2*^ΔSP^ transformant.

To investigate the cellular localization of MoGls2 in *M*. *oryzae*, the complemented transformant Δ*Mogls2/MoGLS2* was observed under a fluorescence microscope. Strong GFP signals were detected in vegetative hyphae, conidium and appressorium of the transformant without a discernable localization pattern. GFP signals were observed in the punctate structures or distributed in conidium and appressorium (Fig 8A). To further clarify the role of the MoGls2 signal peptide, a signal peptide deletion construct pYF11::*MoGLS2*^ΔSP^::GFP was made and transformed into the Δ*Mogls2* mutant. The resulting GFP transformant Δ*Mogls2/MoGLS2*^ΔSP^ was observed under a fluorescence microscope and phenotypic analyzed. Similar to the Δ*Mogls2* mutant, Δ*Mogls2/MoGLS2*^ΔSP^ showed defects in vegetative growth, asexual development, stress response, and virulence (Fig 8B, S2 Table.). However, the localization pattern of *MoGLS2*^ΔSP^::GFP showed no remarkably changes in vegetative hyphae, conidium, and appressorium compared to the Δ*Mogls2/MoGLS2* transformant (Fig 8C), suggested that the signal peptide is indispensable for the function but not cellular localization of MoGls2 in *M*. *oryzae*.

## Discussion

In this study, we identified and characterized MoGls2 in *M*. *oryzae*, which is a homolog of Gls2 in *S*. *cerevisiae* [17]. By bioinformatics analysis found that the sequence of MoGls2 contains highly conserved domains, and showed highly sequence identity among fungi, implicating that Gls2 was a conserved protein during evolution and might have similar functions in different organisms. Expression profile revealed that MoGls2 was induced at early stage, the expression increased at 5 hpi, and reached to highest level at 48 hpi, implicating that MoGls2 likely plays a role during appressorial formation and infection. Target deletion of *MoGLS2* results in decreased growth rate on different media but no morphology change, suggested the growth defect of the Δ*Mogls2* mutant was not nutrient dependent. However, despite the pivotal function of glucosidase II in glycoprotein processing and maturation, Δ*gls2* mutants in *S*. *cerevisiae* and *S*. *pombe* shown no apparent growth defects [13,30], which was inconsistent with the Δ*Mogls2* mutant. These indicated that Gls2 displayed function differentiation in different organisms, though they were conserved in amino acid sequences and protein structures.

Conidium and appressorium are key structures in *M*. *oryzae* disease cycle. Conidium attaches to the surface of rice and form appressorium, then starts the infection cycle [3]. We found *MoGLS2* knock out lead to more production of conidia, and the expression of three conidiation related genes (*MoCOS1*, *MoHOX2* and *MoSTUA*) was significantly increased, indicating *MoGLS2* was a negative regulator of conidiation and might have direct or indirect relationship with these three genes. Meanwhile, appressorium formation was delayed in the Δ*Mogls2* mutant at 2 and 4 hpi, indicating that MoGls2 was important for appressorium formation and probably has a critical role during the early stage of infection, which is consistent with the expression profile data and the result that the mutant has defect in appressorial penetration. Infectious hyphal growth of the Δ*Mogls2* mutant showed defects in the rice sheath cells at 26 hpi, this result is also consistent with the expression profile of *MoGLS2* during infection stages. In addition, the mutant was blocked in perithecia production; this gives us a clue that MoGls2 was a protein of the rice blast fungus with pleiotropic roles.

Cell wall is important for maintaining cell morphogenesis, and presents protection from external stresses [31]. In *M*. *oryzae*, the cell wall integrity was essential for penetration and infectious growth of the pathogen [23,32]. The Δ*Mogls2* mutant showed defects in cell wall integrity because it showed increased sensitivity to cell wall stresses and less sensitive to lysing enzyme. However, chitin contribution and chitin content was not changed in the mutant, though *MoCHS7* was significantly up-regulated. This is reasonable since *MoCHS7* was not a major component for chitin synthesis in *M*. *oryzae* [33]. In *S*. *cerevisiae*, glucosidases are essential for cell wall 1,6-*β*-glucan synthesis [17], indicating that MoGls2 might also participate in glycoprotein and glucan synthesis. Besides, the structure of sugar chains of the fungal cell wall may be crucial for establishment of a functioning interface with the host plant [34], the cell wall defect of the Δ*Mogls2* mutant may also due to the glycoprotein and glucan metabolic disorders, thereby affects stress response and infection of the rice blast fungus. Plant pathogenic fungi deploy secreted effectors to suppress host immunity responses and successfully establish infection on plants [35]. Recently report found that the effector Slp1 should be *N*-glycosylated by an α-1,3-mannosyltransferase and is required to evade host innate immunity in *M*. *oryzae* [20]. However, whether MoGls2 has similar functions to *N*-glycosylation of effectors needs further studies.

In summary, a glycoside hydrolase MoGls2 was identified and highlighted the role in the development and infection in *M*. *oryzae*. We believe that the results presented here are valuable for understanding *N*-linked glycosylation in plant pathogens. Further insights into the role of Gls proteins will be provided by identification and characterization of other components of glycoside hydrolase family during the development and infection of this phytopathogen.

## Supporting Information

S1 Fig Domain prediction of MoGls2 and phylogenetic analysis of Gls2 proteins. (A) Prediction of domains of MoGls2 in SMART website (http://smart.embl-heidelberg.de/). (B) Phylogenetic analysis of MoGls2 and other Gls2 homologues from other organisms by a Clustal_W 1.83 program. Sequences alignments were performed using the Clustal_W program and the calculated phylogenetic tree was viewed using the Mega5.0 Bata program. All of the Gls2 proteins were downloaded from the NCBI database and their accession numbers are listed as following: *Magnaporthe oryzae* (XP_003711051.1), *Aspergillus oryzae* (XP_001822711.1), *Trichoderma reesei* (XP_006964677.1), *Fusarium oxysporum* (EXK23748.1), *Beauveria bassiana* (KGQ08842.1), *Verticillium dahlia* (XP_009653986.1), *Neurospora crassa* (XP_961163.3), *Penicillium roqueforti* (CDM29671.1), *Schizosaccharomyces pombe* (NP_593490.1), *Saccharomyces cerevisiae* (AJP84516.1) and *Gaeumannomyces graminis* (XP_009223395.1).
(TIF)

S2 Fig Deletion of *MoGLS2* gene in *M*. *oryzae*. (A) Schematic illustration for *MoGLS2* targeted gene replacement. (B) The *ΔMogls2* mutant was verified by Southern blot analysis. Genomic DNA of wild-type Guy11 and the *ΔMogls2* mutant were digested with *EcoR* V and separated in a 1% agarose gel. The DNA was hybridized with probe 1 and probe 2, respectively.
(TIF)

S3 Fig Measurement of appressorium turgor and detection glycogen accumulation. **(A)** Cytorrhysis assay using a series of concentrations of glycerol (1–4 M). For each glycerol concentration, at least 100 appressoria were observed and the number of collapsed appressoria was counted. (B) Monitoring glycogen accumulation during appressorial maturation with staining solution containing 60 mg of KI and 10 mg of I_2_ per milliliter of distilled water. The experiments were repeated three times.
(TIF)

S4 Fig Observation of chitin distribution and measurement chitin content in the *ΔMogls2* mutant. (A) Wild-type and mutant hyphae were stained with 10 mg/mL CFW for 5 min in darkness and photographed. (B) GlcNa determination by fluorimetric Morgan–Elson method shows no chitin content change in the *ΔMogls2* mutant. Data comprise three independent experiments with triple replications.
(TIF)

S1 Table Primers used in this study. (DOC)

S2 Table Inhibition rate of the wild type, *ΔMogls2*, Δ*Mogls2/MoGLS2*^Δsp^ and Δ*Mogls2/MoGLS2* transformant on different stresses. (DOCX)
