# Correction: Glycoside Hydrolase MoGls2 Controls Asexual/Sexual Development, Cell Wall Integrity and Infectious Growth in the Rice Blast Fungus

**DOI:** 10.1371/journal.pone.0186552

**Published:** 2017-10-12

**Authors:** Mengying Li, Xinyu Liu, Zhixi Liu, Yi Sun, Muxing Liu, Xiaoli Wang, Haifeng Zhang, Xiaobo Zheng, Zhengguang Zhang

Fig 5a is incorrect. The strain names, Guy11, Δ*Mogls2*, Δ*Mogls2/MoGLS2* are incorrectly switched with the incubation times, 2 h, 4 h, and 6 h. Please see the correct Fig 5a here.

**Fig 5:**
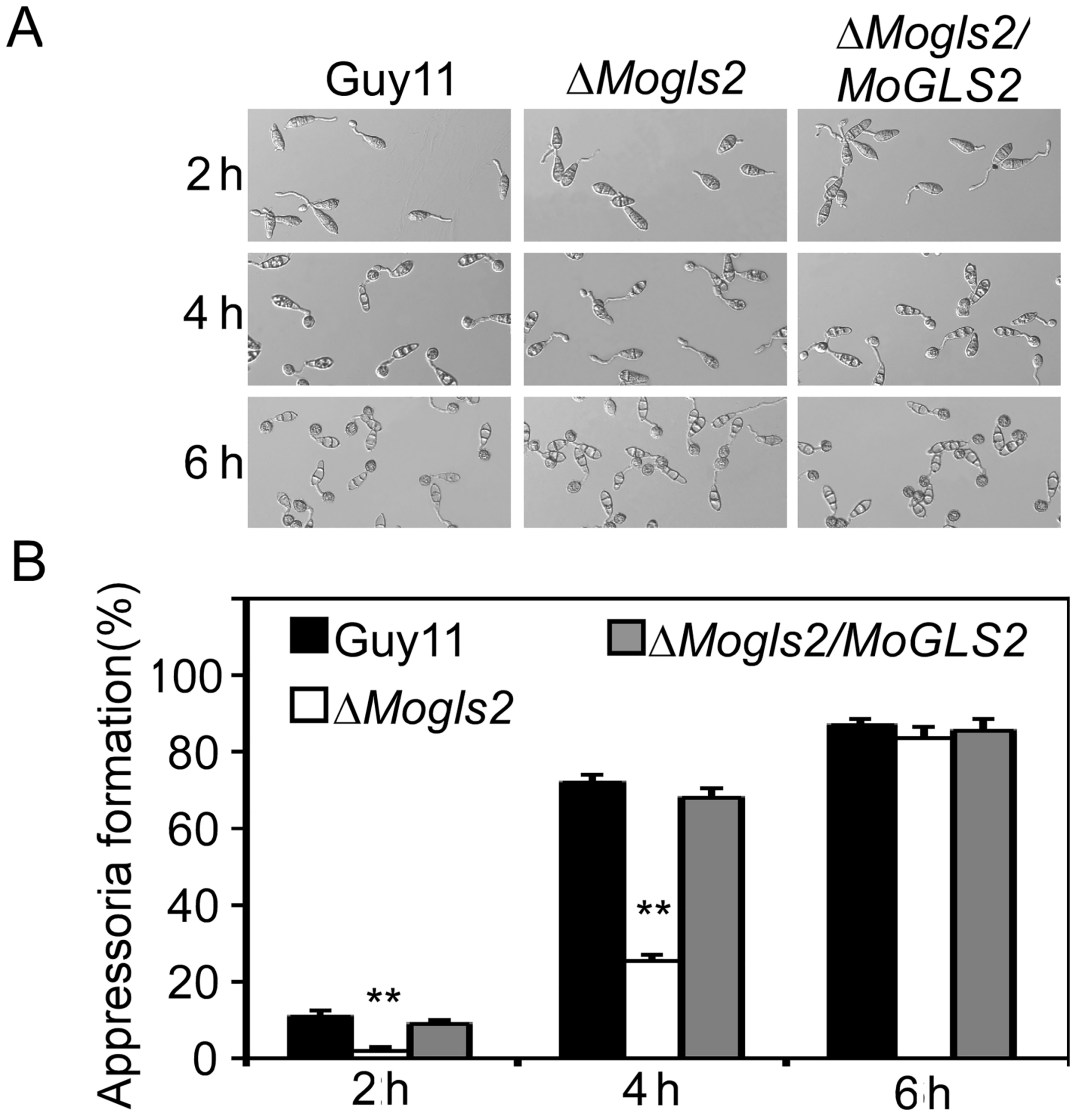
Conidial germination and appressorium formation of the *ΔMogls2* mutant. (A) Conidial germination and appressorium formation was observed at 2, 4 and 6 h on hydrophobic surfaces under a microscope. (B) Statistical analysis of the percentage of appressorium formation at indicated time courses. Error bars are standard deviations and asterisks represent significant differences with *P*<0.01(**). The experiment was replicated three times.
