# Supplementary figures and images for: Glycoside Hydrolase MoGls2 Controls Asexual/Sexual Development, Cell Wall Integrity and Infectious Growth in the Rice Blast Fungus
Source: PLoS One. 2016 Sep 8;11(9):e0162243. doi: 10.1371/journal.pone.0162243 (PMC5015852; doi:10.1371/journal.pone.0162243)

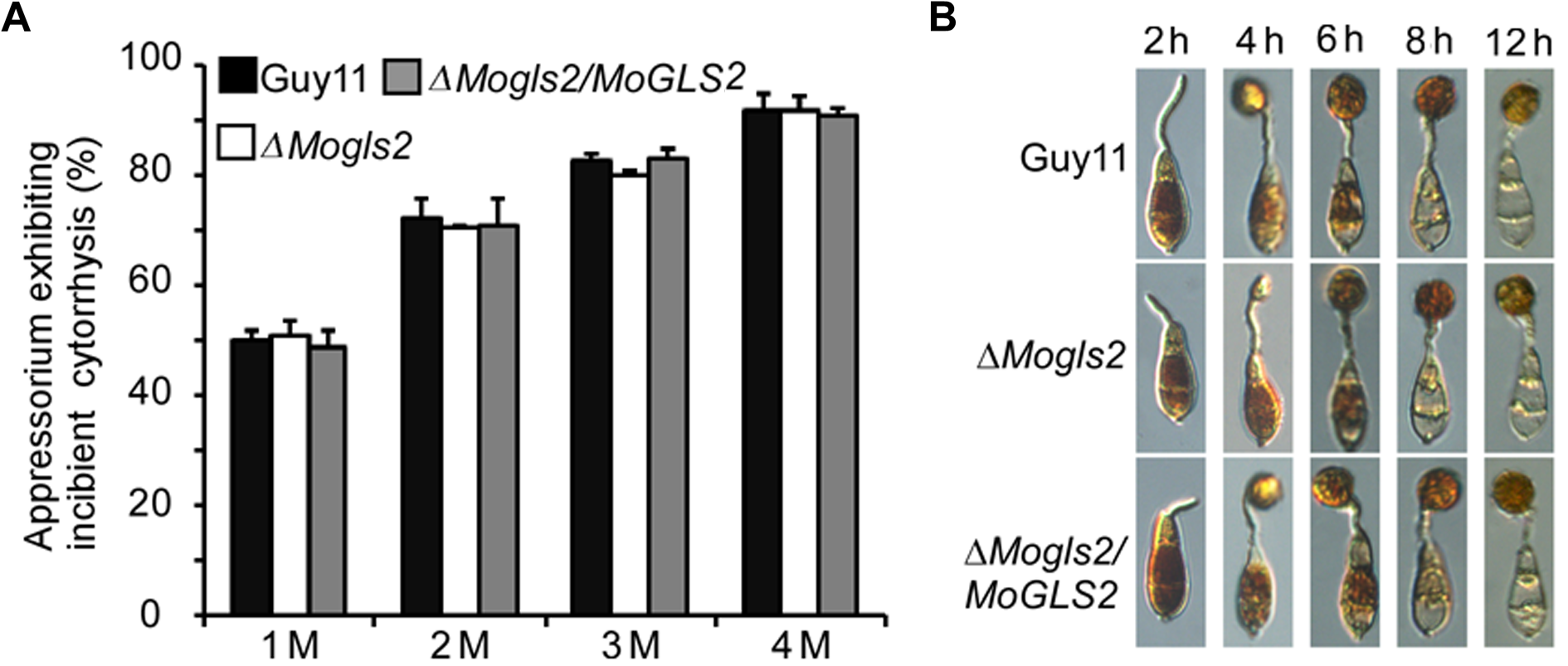

Supplement: S3 Fig — (A) Cytorrhysis assay using a series of concentrations of glycerol (1–4 M). For each glycerol concentration, at least 100 appressoria were observed and the number of collapsed appressoria was counted. (B) Monitoring glycogen accumulation during appressorial maturation with staining solution containing 60 mg of KI and 10 mg of I2 per milliliter of distilled water. The experiments were repeated three times. (TIF) [file pone.0162243.s003.tif]

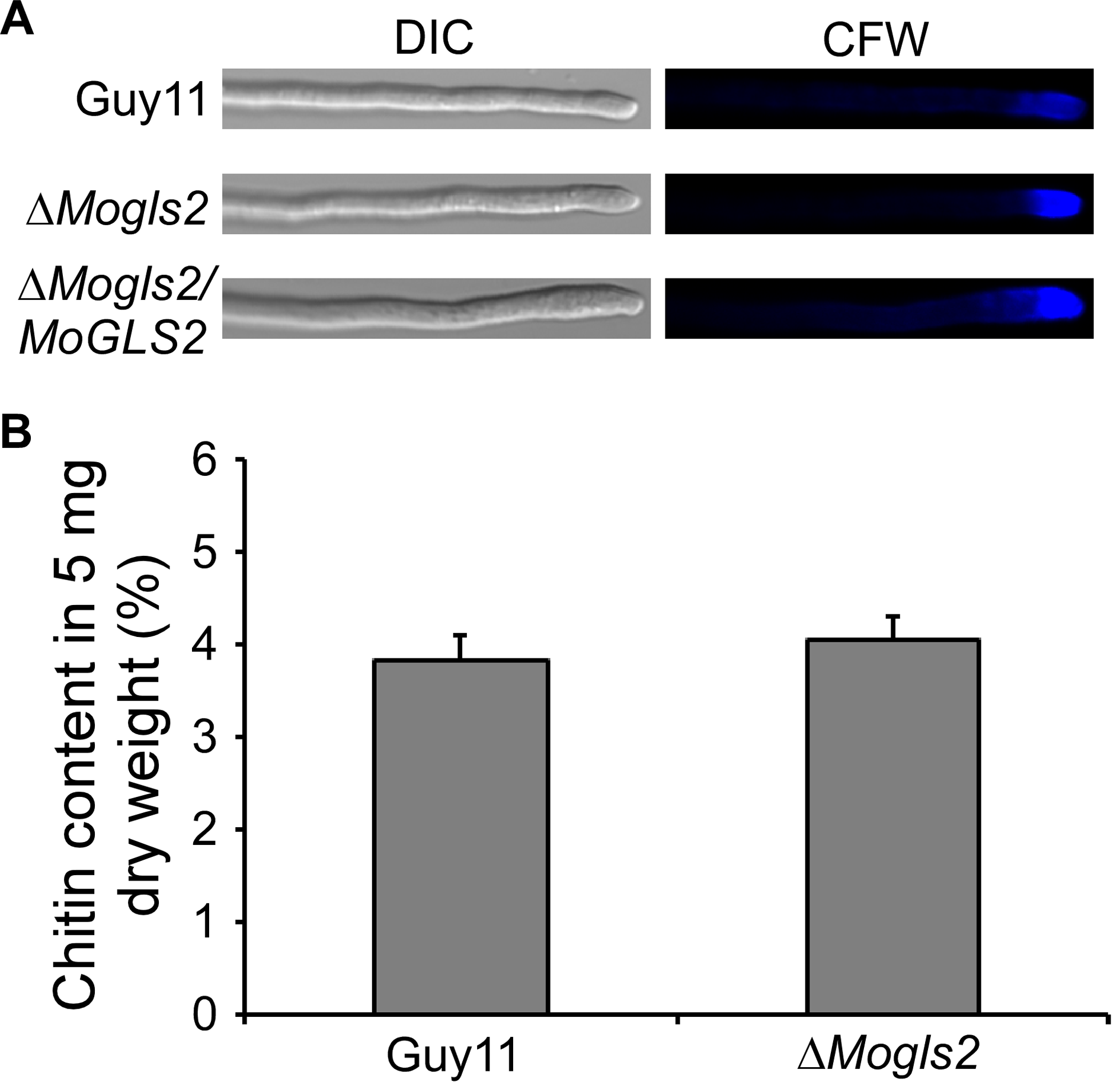

Supplement: S4 Fig — (A) Wild-type and mutant hyphae were stained with 10 mg/mL CFW for 5 min in darkness and photographed. (B) GlcNa determination by fluorimetric Morgan–Elson method shows no chitin content change in the ΔMogls2 mutant. Data comprise three independent experiments with triple replications. (TIF) [file pone.0162243.s004.tif]
