# Supplementary material for: Glycoside Hydrolase MoGls2 Controls Asexual/Sexual Development, Cell Wall Integrity and Infectious Growth in the Rice Blast Fungus
Source: PLoS One. 2016 Sep 8;11(9):e0162243. doi: 10.1371/journal.pone.0162243 (PMC5015852; doi:10.1371/journal.pone.0162243)
Supplement: S1 Table — (DOC) [file pone.0162243.s005.doc]

**Table S1.** Primers used in this study

| **Primer name** | **Sequence (5’-3’)** | **Remark** |
| --- | --- | --- |
| MGG_08623F1 | TAACTCGAGCACGTCTGCTCAGTAGACATT | amplify *MoGLS2* 5’ flank sequence |
| MGG_08623F2 | TAAGATATCATGTATTCTTTAGCCGCAGG | amplify *MoGLS2* 5’ flank sequence |
| MGG_08623F 3 | TAAACTAGTATGGGACGGATATTGGTCCG | amplify *MoGLS2* 3’ flank sequence |
| MGG_08623F 4 | TAATCTAGACAGCGCCTCGAATCCTTCTA | amplify *MoGLS2* 3’ flank sequence |
| MGG_08623P1-F | CAAAGAGCGCTACAATGAAGCT | validation of *MoGLS2* deletion |
| MGG_08623P1-R | GCTCGTTGTAGTTACCATCAC | validation of *MoGLS2* deletion |
| MGG_08623BY-F | TGGGGAGCTGTATTAGAAGTC | validation of *MoGLS2* deletion |
| HphR | CATTGATGTGTTGACCTCC | validation of *MoGLS2* deletion |
| MGG_08623GFP-1 | ACTCACTATAGGGCGAATTGGGTACTCAAATTGGTT GGTCGTCTCAACTTCAATG | *MoGLS2* complementation |
| MGG_08623GFP-2 | CACCACCCCGGTGAACAGCTCCTCGCCCTTGCTCAC CAGTGCTAAAGAAACCTTCC | *MoGLS2* complementation |
| 08623GFP1 | ACTCACTATAGGGCGAATTGGGTACTCAAATTGGTT GGTCGTCTCAACTTCAATG | *MoGLS2*^ΔSP^ complementation |
| 08623dwzq2 | TTACAAAATG GTCAAGGAGCATGACTTCAA | *MoGLS2*^ΔSP^ complementation |
| 08623dwzq1 | GCTCCTTGACCATTTTGTAAATATGTATTC | *MoGLS2*^ΔSP^ complementation |
| 08623GFP2 | CACCACCCCGGTGAACAGCTCCTCGCCCTTGCTCAC CAGTGCTAAAGAAACCTTCC | *MoGLS2*^ΔSP^ complementation |
| qRT-MGG_08623p1 | TTCTACACACACCCGTCCG | quantitative RT-PCR analysis *MoGLS2* |
| qRT-MGG_08623p2 | CACCGTTTCCTTGTCCTTC | quantitative RT-PCR analysis *MoGLS2* |
| Rubq1 LL | GTGGTGGCCAGTAAGTCCTC | qRT-PCR Primer of Rice *ACTIN* |
| Rubq1 RR | GGACACAATGATTAGGGATCA | qRT-PCR Primer of Rice *ACTIN* |
| 28S rDNA LL | TACGAGAGGAACCGCTCATTCAGATAATTA | qRT-PCR Primer of *M. oryzae* 28S rDNA |
| 28S rDNA RR | TCAGCAGATCGTAACGATAAAGCTACTC | qRT-PCR Primer of *M. oryzae* 28S rDNA |
| ACTIN | CCATGTACCCTGGTCTTTCG | qRT-PCR Primer of *ACTIN* |
| ACTIN | TTCGAGATCCACATCTGCTG | qRT-PCR Primer of *ACTIN* |
| qRTCOS1F1 | CCCTCAGCCCACATACAACT | qRT-PCR Primer of *MoCOS1* |
| qRTCOS1F2 | AGCCTTCGCTCGATACTGAA | qRT-PCR Primer of *MoCOS1* |
| qRTCOM1F1 | ACCGATTCTGACGAATCCAG | qRT-PCR Primer of *MoCOM1* |
| qRTCOM1F2 | CTGGAACTGCTGTCCTCCTC | qRT-PCR Primer of *MoCOM1* |
| qRTCON7F1 | GCAAGAAGTGCGTTCAAACA | qRT-PCR Primer of *MoCON7* |
| qRTCON7F2 | TCTCCACTGCTGCCACTATG | qRT-PCR Primer of *MoCON7* |
| qRTCON2F1 | GGAGCCGAAAACATCAACAT | qRT-PCR Primer of *MoCON2* |
| qRTCON2F2 | GTTGGTTGGTCCATGCTCTT | qRT-PCR Primer of *MoCON2* |
| qRTHox2F1  qRTHox2F2 | CGATAATTGCTCCCACACCT  GAAGGAGTCGGTGGTGACAT | qRT-PCR Primer of *MoHOX2*  qRT-PCR Primer of *MoHOX2* |
| qRTStuAF1 | CAACATGGGCAGCTCTGATA | qRT-PCR Primer of *MoSTUA* |
| qRTStuAF2 | CCTGCATGCTTTGTAGCGTA | qRT-PCR Primer of *MoSTUA* |
| qRTCHS1F  qRTCHS1R  qRTCHS2F  qRTCHS2R  qRTCHS3F  qRTCHS3R  qRTCHS4F  qRTCHS4R  qRTCHS5F  qRTCHS5R  qRTCHS6F  qRTCHS6R  qRTCHS7F | TCAACGACGAGGAGAAGCC  GTAATCGCAACAGCCAAGA  TCCACGACCTTTGCCATCA  CGCTTTTGCTTCCGCGACT  CGGAAACCAAGGAACAGCG  CAGGGAACAACCAAGAACCAC  TCGAGGGAAAATGTAACGG  TACTGCTGCTGGTGATGGT  CCGTGTTGATGGAGGTTGA  GATCTGGCGGTCGAGGAAT  GAACGGCAGATTTGATGAC  ACAAGAGTGCTTCGGTGGC  GACATTGAGCTGGAGATTGG | qRT-PCR Primer of *MoCHS1*  qRT-PCR Primer of *MoCHS1*  qRT-PCR Primer of *MoCHS2*  qRT-PCR Primer of *MoCHS2*  qRT-PCR Primer of *MoCHS3*  qRT-PCR Primer of *MoCHS3*  qRT-PCR Primer of *MoCHS4*  qRT-PCR Primer of *MoCHS4*  qRT-PCR Primer of *MoCHS5*  qRT-PCR Primer of *MoCHS5*  qRT-PCR Primer of *MoCHS6*  qRT-PCR Primer of *MoCHS6*  qRT-PCR Primer of *MoCHS7* |
| qRTCHS7R | CGCCGCTGTTGCTGTTGTT | qRT-PCR Primer of *MoCHS7* |
