# Supplementary material for: Glycoside Hydrolase MoGls2 Controls Asexual/Sexual Development, Cell Wall Integrity and Infectious Growth in the Rice Blast Fungus
Source: PLoS One. 2016 Sep 8;11(9):e0162243. doi: 10.1371/journal.pone.0162243 (PMC5015852; doi:10.1371/journal.pone.0162243)
Supplement: S2 Table — (DOCX) [file pone.0162243.s006.docx]

Table S2. Inhibition rate of the wild type, *∆Mogls2*, ∆*Mogls2/MoGLS2*^∆sp^ and ∆*Mogls2/MoGLS2* transformant on different stresses.

| Strain | Inhibition rate (%) | | | | | |
| --- | --- | --- | --- | --- | --- | --- |
|  | NaCl | KCl | Sorbitol | CFW | CR | SDS |
| Guy11 | 76.3 ± 3.2 | 79.1±4.0 | 62.7±3.8 | 60.7 ±3.9 | 22.9±1.2 | 78.8±3.9 |
| *∆Mogls2* | 57.3 ± 1.1^*^ | 68.2±3.2^*^ | 28.5± 1.9^*^ | 83.4± 4.7^*^ | 48.8±4.0 ^*^ | 88.5± 3.3^*^ |
| ∆*Mogls2/ MoGLS2*^∆sp^ | 59.6 ± 2.5^*^ | 70.7± 5.9^*^ | 27.6± 0.8^*^ | 81.1 ±2.1^*^ | 46.3± 2.8^*^ | 90.3± 5.0^*^ |
| ∆*Mogls2/MoGLS2* | 73.1 ± 5.8 | 76.2±6.3 | 63.2 ±3.1 | 65.3±3.2 | 20.8±1.7 | 76.0±1.3 |

Asterisks represent significant differences with *P*<0.01(**).
